# Prevalence of sexual experience among Korean adolescent: age-period-cohort analysis

**DOI:** 10.4178/epih.e2020008

**Published:** 2020-03-03

**Authors:** Yongho Jee, Gyuyoung Lee

**Affiliations:** 1 Department of Public Health, Graduate School of Public Health, Seoul National University, Seoul, Korea; 2 Red Cross College of Nursing, Chung-Ang University, Seoul, Korea

**Keywords:** Sexual behavior, Adolescent, Age, Period, Cohort effect

## Abstract

**OBJECTIVES:**

Since exposure to sexual content and early sexual initiation among adolescents have become serious social issues in Korea, an in-depth analysis of trends in the prevalence of sexual experience among Korean adolescents is necessary to project the trends and policies required for the next 10 years. The objective of this study was to identify the contributions of age, period, and birth cohort effects on the prevalence of sexual experience in Korean adolescents.

**METHODS:**

We analyzed age-specific, period-specific, and birth cohort–specific trends in the prevalence of sexual experience among 911,502 adolescents (469,593 boys, 51.5%; 441,909 girls, 48.5%) aged 12 years to 17 years from the 2006 to 2017 Korean Youth Risk Behavior Web-based Survey. Joinpoint regression analysis was conducted to examine significant changes in the prevalence of sexual experience and to find the optimal number and location of places where trends changed.

**RESULTS:**

The prevalence of sexual experience generally increased with age in all periods in both boys and girls. In boys, the prevalence of sexual experience increased in recent periods, especially in the age group of 12-13 years, while the prevalence of sexual experience decreased in the age group of 16-17 years. In girls, the age group of 12-13 years showed an increased prevalence of sexual experience in recent periods. However, the prevalence showed a decreasing trend in the age group of 16-17 years.

**CONCLUSIONS:**

In boys and girls, sexual experience increased with age, although this tendency has slowed in recent cohorts. Therefore, early sex education is needed.

## INTRODUCTION

The beginning of sexual intercourse is a key social development transition in adolescence with regard to physical maturation, cognitive development, increased awareness and appreciation of one’s body, strengthening of individual and gender identity, and formation of sexual relations [1-3]. The prevalence of sexual experience (defined as ever having had sex) is a common index measuring sexual activity at the population level. In the 20th century, most industrialized nations have experienced a decline in the age of sexual initiation among adolescents from cohorts born in the 1920s to those born in the 1970s [4]. However, the median age of sexual debut and related social norms vary by region and culture, and no universal trend has been found towards earlier sexual intercourse [5].

Patterns of sexual behaviors are converging across developed countries. In other words, variation in the timing of sexual initiation has decreased within and between countries. In the United States, although the prevalence of sexual experience among adolescents from 1991 to 1997 decreased by 11%, the age at first intercourse continues to decline [6]. Korean society has traditionally been based on Confucian norms, according to which premarital sexual intercourse and early initiation of sexual intercourse are regarded as taboo [7]. Adolescents in Europe also showed a reduction in age of sexual initiation starting in the 1950s. Early sexual intercourse is typically defined as sexual initiation before the ages of 13 or 14 [8]. Its frequency varies by gender and race/ethnicity, with higher rates among boys and black or Hispanic teens [9-11]. According to a recent report from the Korea Centers for Disease Control and Prevention (KCDC), the prevalence of having experienced sexual intercourse was 4.8% among 31,362 adolescents in 2016 [7]. Although this rate is lower than that of Western countries, adolescents’ experiences of sexual intercourse are still an important topic because numerous studies have reported associations between earlier sexual activity and negative physical and mental health outcomes such as the risk of sexually transmitted infections and depressive symptoms [12-15].

Life course theory is a prospective view that looks at individuals’ or cohorts’ life experiences, including analyses that span generations, to find clues to future patterns of health and disease, considering that both past and present experiences are shaped by the socioeconomic and cultural context [16]. In the framework of the life course theory, adolescents are in a transitional period before becoming adults. Therefore, our study is meaningful in that we provide basic data for predicting patterns of sexual behavior among adults in their 30s and 40s in the future by analyzing patterns of sexual behavior among adolescents. Although the prevalence of sexual experience among Korean adolescents is relatively low compared to that of adolescents in Western countries, younger generations are more prone to have early sexual experiences than older generations owing to the emergence of an information-oriented society in which adolescents are exposed to numerous sources of unfiltered content and media [17]. However, most studies on the prevalence of sexual experience among Korean adolescents were cross-sectional studies [18,19]. Such studies are unsuitable for analyzing the age-specific or birth cohort-specific prevalence of sexual experience [20,21].

The age-period-cohort (APC) model has been widely used to overcome the limitations of cross-sectional data [22-24]. Previous studies have used the APC model not only for analyzing long-term trends of mortality and the incidence of chronic disease [22,25,26], but also for analyzing lifestyle patterns such as smoking in association with death rates [27,28]. By analyzing the independent effects of age, period, and birth cohort on changing trends in the prevalence of sexual experience among Korean adolescents aged 12-17 years, our study aimed to provide evidence that could be used to develop intervention strategies for improving adolescents’ sexual health and to project the prevalence of sexual experience among adolescents into the future. Since the age at first sexual experience is becoming younger, we conducted an APC analysis to test our hypothesis that the prevalence of sex experience has increased among older age groups, in recent periods, and in younger cohorts.

## MATERIALS AND METHODS

### Subjects

The KCDC established the Korea Youth Risk Behavior Web-based Survey (KYRBS) in 2005. The KYRBS is an ongoing annual nationwide cross-sectional survey that investigates health-risk behaviors among middle-school and high-school students. It provides data for the development and evaluation of school health policies and programs in Korea [29]. Our study examined 911,502 adolescents (boys: 469,593 [51.5%]; girls: 441,909 [48.5%]) aged 12-17 years in the KYRBS data spanning from 2006 to 2017. In this study, adolescents who were 18 years old were included in the 17-year-old age group. More specifically, the subjects comprised, 71,093 adolescents in 2006, 73,836 adolescents in 2007, 74,451 adolescents in 2008, 74,192 adolescents in 2009, 72,623 adolescents in 2010, 75,205 adolescents in 2011, 73,850 adolescents in 2012, 72,047 adolescents in 2013, 71,638 adolescents in 2014, 67,671 adolescents in 2015, 65,212 adolescents in 2016, and 61,861 adolescents in 2017.

### Study variables

To identify trends in the prevalence of sexual experience in Korean adolescents using an APC model, we extracted the following variables from the annual survey data: period, age, gender, number of population, number of adolescents who had ever experienced sexual intercourse, and weighted values.

### Statistical analysis

To investigate trends in the age-specific, period-specific, and birth cohort–specific prevalence of sexual experience for both boys and girls who participated in the KYRBS, we requested and obtained the KYRBS raw data for years of 2006 to 2017. We divided age into 3 groups (12-13, 14-15, and 16-17 years) for each period. Six periods were defined (2006-2007, 2008-2009, 2010-2011, 2012-2013, 2014-2015, and 2016-2017), and participants were divided into 6 birth cohorts (1992-1993, 1994-1995, 1996-1997, 1998-1999, 2000-2001, and 2002-2003) to analyze the prevalence of sexual experience in each birth cohort. We used age-adjusted figures for the prevalence of sexual experience based on the 2005 national population to eliminate differences caused by changes in age distribution.

### Age-period-cohort analysis

Changes in the prevalence of sexual experience from 2006 to 2017 were examined in terms of age, period-cohort, age-period, and age-cohort effects. Furthermore, the independent effect of each factor on sexual experience was estimated via APC analysis. The problem of identification caused by collinearity among age, period, and cohort (age+cohort= period) [23,24] was solved by using the intrinsic estimator (IE) method. The fit of the APC model with different combinations of age, period, and cohort was analyzed using the Akaike information criterion and differences in deviance values. For the IE analysis, the apc_ie package of Stata version 13.1 (StataCorp., College Station, TX, USA) was used. All analyses were performed separately for boys and girls. All statistical analyses were performed using SAS version 9.4 (SAS Institute Inc., Cary, NC, USA) and Stata 13.1 (StataCorp.). The significance level was set at p-value < 0.05.

### Joinpoint analysis

Joinpoint regression analysis was conducted to examine significant changes in the prevalence of sexual experience and find the optimal number and location of places where trends changed [30]. We used the 3.5.3 version of Joinpoint software developed by the Surveillance Research Program of the United States National Cancer Institute, which was based on the Poisson assumption.

### Ethics statement

Data from the KYRBS survey are made publicly available through the KYRBS website (http://www.cdc.go.kr/yhs/). Thus, ethical approval was not required for this study. All participants provided written informed consent.

## RESULTS

**Figure 1.**
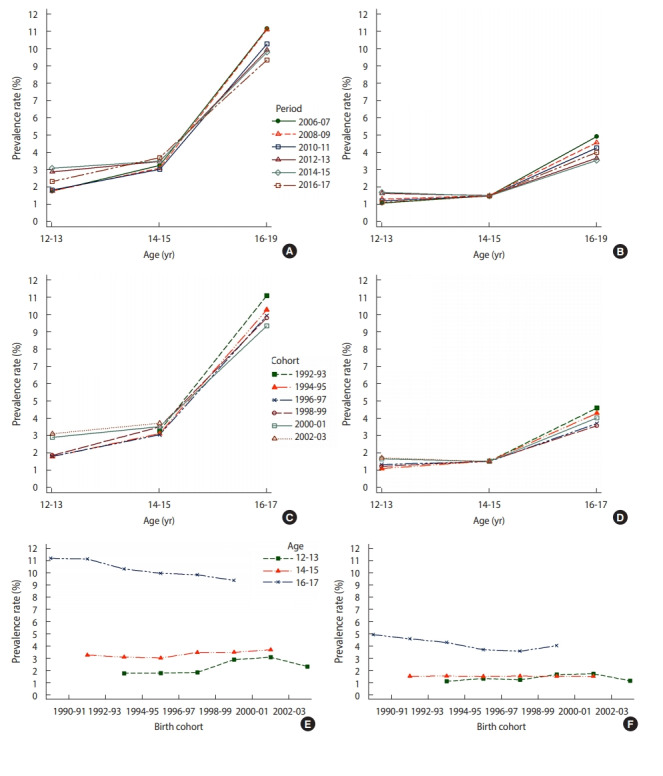
Age-period-cohort effects on the age-specific prevalence of sexual experience from 2006 to 2017 in boys and girls. Age-specific prevalence of sexual experience by period in boys (A) and girls (B), age-specific prevalence of sexual experience by birth cohort in boys (C) and girls (D), and birth cohort–specific prevalence of sexual experience by age in boys (E) and girls (F).

Table 1 shows the sex-specific, age-specific, and period-specific prevalence of sexual experience among subjects. The prevalence of sexual experience generally increased by age in all periods in both boys and girls. In boys, the prevalence of sexual experience increased in recent periods, especially in the age group of 12-13 years, while the prevalence of sexual experience decreased in the age group of 16-17 years. In girls, the age group of 12-13 years showed an increased prevalence of sexual experience in recent periods. However, the prevalence showed a decreasing trend in the age group of 16-17 years. Figure 1A and 1B depicts the age-specific prevalence of sexual experience in boys and girls by period. The age-specific prevalence of sex experience in the age group of 12-13 was higher in 2012-2015 than in other periods. However, the age group of 16-17 years showed the highest prevalence in 2006-2007. Figure 1C and 1D shows the birth-cohort specific prevalence of sexual experience by age groups. In general, the prevalence of sexual experience increased with age in the older birth cohorts. In boys, the age group of 16-17 years showed a decrease in the prevalence of sexual experience in increasingly younger cohorts, while the prevalence of sexual experience increased in younger age groups. In girls, all age groups except the group of 12-13 years showed a trend for the prevalence of sexual experience to decline in increasingly younger cohorts. However, the rate of decline slowed in recent cohorts.

**Figure 2.**
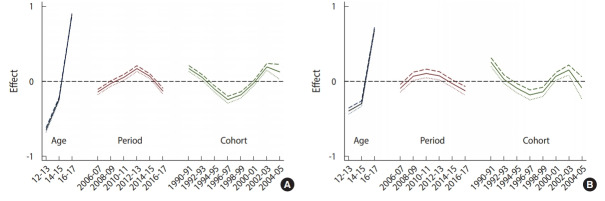
Age-period-cohort analysis of the prevalence of sexual experience in Korean boys (A) and girls (B).

**Figure 3.**
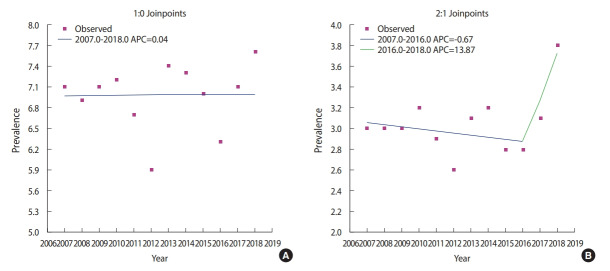
Joinpoint analysis of the prevalence of sexual experience in Korean youth. APC, age-period-cohort.

Table 2 shows the results of various models explaining the prevalence of sexual experience. Of these models, the APC and age-cohort models best explained the prevalence of sexual experience in both boys and girls. Figure 2 presents a schematic diagram of the APC model shown in Table 2. In boys, the prevalence of sexual experience rapidly escalated with age. The prevalence of sexual experience steadily increased from 2006 to 2011, and declined in the birth cohorts prior to 1996 and 1997, before steadily increasing in recent cohorts. The results for girls were similar to those for boys in general. The overall increase by period was somewhat more stable in girls than in boys. In the Joinpoint analysis, changes in linear trends were detected (Figure 3). The prevalence of sexual experience among Korean youth decreased from 2007 to 2016, but increased from 2016 to 2018 in girls.

## DISCUSSION

Our study aimed to identify the independent effects of age, period, and cohort on trends in the prevalence of sexual experience in adolescents aged 12-17. The prevalence of sexual experience increased with age. Especially in a relatively recent cohort (1998- 1999), the prevalence of sexual experience was high in the youngest age group (12-13 years).

In our study, boys were found to be more likely to engage in sexual intercourse than girls. This finding is consistent with previous studies from other countries, including the Netherlands [31] and the United States [32-34]. However, this discrepancy may be explained by the social context emphasizing chastity for women, which may lead girls to under-report their sexual experience, whereas boys may tend to over-report their sexual experience since traditional norms regard sexual prowess as a crucial component of masculinity [35].

It has been previously found that boys’ sexual attitudes are influenced primarily by individual factors including curiosity, while females’ sexual attitudes are influenced more by family-related factors such as parental attitudes [36]. Therefore, from a social perspective, rapid exposure to massive amounts of sexually explicit media may be associated with the increased prevalence of sexual experience among boys, while the increasing prevalence of the nuclear family structure might be associated with the increased prevalence of sexual experience among girls [37].

From a demographic perspective, the increasing rate of later marriage in most countries has led to an increase in premarital sex. The prevalence of premarital sex is generally higher in developed countries than in developing countries. Furthermore, it is generally higher in men than in women [38-40]. The average age of first marriage in Korea was reported to be 32.94 years for men and 30.24 years for women [41]. Therefore, increased early exposure to sexual content and late marriage might be associated with the overall increasing prevalence of sexual experience in younger generations.

Since sexual activity is commonly initiated during adolescence, it is often accepted as a normative part of the transition to adulthood. However, sexual activity can lead to negative consequences such as sexually transmitted infections and unwanted pregnancies [42]. Sexual activity in adolescents is more dangerous than sexual activity in adults, not only due to adolescents’ relative physical, emotional, and cognitive immaturity, but also due to their tendency to engage more frequently in risky behaviors such as unprotected intercourse [43]. A study of data from a European population reported that in Western, Central, and Eastern Europe, 40% of adolescents who have had intercourse did not use condoms when they most recently engaged in sexual intercourse [42]. Links between early sex and other risky behaviors including smoking, alcohol drinking, and drug use have also been reported in previous studies [44,45].

Existing studies have suggested that the trend toward a younger age at sexual initiation in general is common. Thus, patterns of adolescents’ sexual behaviors appear to be becoming increasingly homogeneous in developed countries [46]. The results from our study using KYRBS are consistent with those of previous studies that showed that the prevalence of sexual experience at age 12 has generally increased in recent cohorts. Moreover, Lee [47] reported that 22.3% of adolescents had their first sexual experience in elementary school, 39.4% in middle school, and 38.2% in high school. This implies that the prevalence of sexual experience in Korean adolescents may continue to increase in the future. This might be due to urbanization, industrialization, and informatization in the past few decades in Korea. These changes might have enabled young generations to be exposed to massive amounts of information through the mass media [37]. Although the prevalence of sexual experience in Korean adolescents is lower than has been reported in data from Western countries, the growth in its prevalence is steep and steady for Korean society, which is strongly Confucian. Findings of previous studies have documented benefits of delaying intercourse, at least into the late teens and ideally into early adulthood [48,49].

Previous findings have suggested the importance of enhancing problem-solving skills, health-promotion behaviors, and appropriate education [50]. Jessor & Jessor [50] observed that adolescents with poor problem-solving skills are more likely to engage in behaviors such as precocious sex. Because problem-solving involves perceiving, processing, and using information regarding oneself, it plays an important role in behavioral health. In general, problem-solving comprises motivation, perceived self-efficacy, and the ability to regulate cognitive, behavioral, and emotional reactions when solving problems. Therefore, rather than merely regulating the sexual experience of adolescents through traditional education that emphasizes chastity, it is important to teach decision-making and problem-solving skills to prevent unwanted sexual behavior and to provide the necessary knowledge for safe sex.

In conclusion, although the prevalence of sexual experience among Korean adolescents from 2006 to 2017 slightly decreased, the prevalence of early sexual experience is increasing. Thus, it is necessary to strengthen sex education in schools to include not only contraception or chastity, but also life skills such as refusal and self-decision-making skills from an early age.

There are several strengths of this study. First, it used representative data from the KYRBS. Second, in terms of methodology, we employed an APC model and joinpoint analysis to analyze long-term trends in the prevalence of sexual experience at various ages. This method allows researchers to analyze age effects; period effects, defined as the social and environmental effects on everyone in a particular period; and birth cohort effects, which only specific birth cohorts experience, as separate risk factors. However, separating these 3 effects is challenged by the statistical collinearity among them. We solved this problem by using the IE method. Since our data on the prevalence of sexual experience were drawn from self-report measurements, the validity of the data has limitations. Thus, the results of this study require cautious interpretation.

## Figures and Tables

**Table 1.** Prevalence of sexual experience according to gender, age, and period from 2006 to 2017 based on the Korea Youth Risk Behavior Web-based Survey

Gender	Age (yr)	Period											
		2006-2007		2008-2009		2010-2011		2012-2013		2014-2015		2016-2017	
		n (%)	SE	n (%)	SE	n (%)	SE	n (%)	SE	n (%)	SE	n (%)	SE
Boys	12-13	197,274 (1.8)	0.001	18,949 (1.8)	0.001	17,561 (1.9)	0.001	19,940 (2.9)	0.001	18,067 (2.0)	0.001	15,858 (1.5)	0.001
	14-15	25,988 (3.3)	0.001	27,642 (3.1)	0.001	26,400 (3.0)	0.001	25,436 (3.5)	0.001	24,631 (3.5)	0.001	22,002 (3.7)	0.001
	16-17	30,668 (11.2)	0.002	31,267 (11.1)	0.002	31,639 (10.3)	0.002	29,075 (9.9)	0.002	28,477 (9.8)	0.002	27,091 (9.4)	0.002
Girls	12-13	16,825 (1.1)	0.001	16,501 (1.2)	0.001	16,709 (1.2)	0.001	18,681 (1.6)	0.001	16,914 (1.7)	0.001	15,123 (1.1)	0.001
	14-15	24,150 (1.5)	0.001	23,765 (1.3)	0.001	24,399 (1.4)	0.001	23,886 (1.1)	0.001	23,454 (1.5)	0.001	21,102 (1.5)	0.001
	16-17	28,024 (4.9)	0.001	30,519 (4.5)	0.001	31,120 (4.2)	0.001	28,879 (3.7)	0.001	27,763 (3.6)	0.001	25,897 (4.0)	0.001

SE, standard error.

**Table 2.** Goodness-of-fit of the APC model for the prevalence of sexual experience among Korean youth

Gender	Model	Log L	AIC	Residual df	Δ AIC	Δ df
Boy	APC	-84.8	11.0	4	0.0	0
	AC	-141.3	16.8	8	5.8	4
	AP	-187.1	21.7	10	10.7	6
	PC	-273.6	31.8	5	20.8	1
	Age	-197.6	22.3	15	11.3	11
Girl	APC	-77.4	10.2	4	0.0	0
	AC	-92.2	11.4	8	1.2	4
	AP	120.8	14.3	10	4.1	6
	PC	-213.8	25.2	5	15.0	1
	Age	-139.7	15.9	15	5.7	11

APC, age-period-cohort; AIC, Akaike information criterion; df, degree of freedom; AC, age-cohort model; AP, age-period model; PC, period-cohort model.
